# Understanding health data social licence: An international comparison of community attitudes towards health data use across Canada and Australia

**DOI:** 10.23889/ijpds.v9i5.1660

**Published:** 2024-09-18

**Authors:** Zoe White, Josie Plachta, Rachel Shipsey

**Affiliations:** 1 Office for National Statistics

## Objective

The Office for National Statistics aim is to create a platform containing a variety of datasets interlinked via data spines. The resource burden linking this many datasets is high, and so research to maximise automation is a key aim of the platform’s methodology. Probabilistic linkage is a standard linkage technique that utilises clerical resolution – a very costly process – to find acceptance threshold parameters. Here, we present research into automating threshold assignment.

Every admin dataset is different, causing the probabilistically matched pairs scores to change, a set threshold cannot be used. Instead, a data-informed threshold must be set.

## Approach

A gold standard linked dataset was linked through Fellegi-Sunter, stopping at the point of finding the probabilistic threshold. To find the threshold, graphs plotting the number of records at each rounded score were created and reviewed, specifically looking at the spread of gold standard links, deterministic links, unique and non-unique probabilistic links.

This review suggested a method of threshold assignment that provides multiple quality levels: a high-precision threshold, and a high-recall threshold. This presentation will showcase these graphs and explain how the two thresholds are calculated.

The 2021 Census is one dataset linked within the gold standard linked dataset. The 2021 Census has high quality and relatively clean data, but is likely not representative of administrative data needed in the data platform. Therefore, next steps are testing this method on other datasets with a variety of quality, characteristics, and types.

